# Introducing *World Journal of Emergency Surgery*

**DOI:** 10.1186/1749-7922-1-1

**Published:** 2006-03-24

**Authors:** Fausto Catena, Ernest E Moore

**Affiliations:** 1 Emergency Surgery Department, St Orsola-Malpighi University Hospital, Bologna, Italy; 2 Trauma Services, Rocky Mountain Regional Trauma Center at Denver Health Medical Center, Colorado, USA; 3 Department of Surgery, Denver Health Medical Center, Colorado, USA; 4 Department of Surgery, University of Colorado Health Sciences Center, Colorado, USA

About a year ago I contacted Professor Moore in Denver and presented the idea of founding an Emergency Surgery Journal for worldwide participation. He replied immediately that it was a very timely concept because a new discipline termed " acute care surgeon " was being developed in the USA to meetthe expanding roleof trauma surgeons, and that he was aware that this construct existed in other countries. In fact, there are no specific peer-reviewed emergency surgery journals, although several journals exist in the field of trauma surgery, including "Injury", "The Journal of Trauma", "The Panamerican Journal of Trauma", "Shock", "Turkish Journal of Trauma and Emergency Surgery " [1].

Emergency surgery is a transverse topic as all surgical specialities include emergency surgery.

Therefore all surgeons have the "problem" of emergency surgery. It is hence important to have a substantial base that provides an ample array of knowledge on all the aspects of this topic from the point of view of every speciality, as not all surgical specialities are present in many hospitals.

*World Journal of Emergency Surgery *(WJES) [2] is a peer-reviewed, open access online journal covering all aspects of research in emergency surgery (traumatic and non-traumatic) and related fields.

As previously stated, emergency surgery is a multidisciplinary super-specialty involving all surgical and emergency medicine specialties; this field concerns all surgeons and physicians around the world. Emergency surgery is divided into traumatic emergency surgery and non-traumatic emergency surgery; both of which, are a fundamental part of the surgeon's daily work. Emergency surgery not only includes general emergency surgery, vascular emergency surgery, and thoracic emergency surgery but also urologic, cardiac, paediatric, musculoskeletal, gynaecological, transplant emergency surgery and all other surgical specialties; all of which, will be covered by WJES.

In the last two months we have sent 1000 surgeons around the world the World Journal of Emergency Surgery "Emergency Surgeon Questionnaire" in regards to the emergency surgery organization [3]. We are going to present the results in a formal research manuscript in WJES, however, it is important to note that 60% of hospitals around the world have Emergency Surgery departments or units. Therefore it is evident that emergency surgery has also an identity in many hospitals with doctors specifically involved in this field.

*World Journal of Emergency Surgery *follows a closed peer review process. The editorial board will assess articles submitted to the journal, and those manuscripts that are deemed suitable for peer review will be assigned to at least two expert reviewers. The Editors-in-Chief will decide on whether to accept or reject a manuscript based on reviewer recommendations. The goal is to make a first decision within four weeks. Accepted articles will be published immediately online with their final citation, and will soon after be listed in PubMed [4].

*World Journal of Emergency Surgery *is published by BioMed Central, an independent publishing house committed to ensuring that peer-reviewed biomedical research is open access – immediately and permanently available online without charge or any other barriers to access.

Traditionally, readers pay to access research articles, either through subscriptions or by paying a fee each time they download an article [5]. Escalating journal subscription charges have resulted in libraries subscribing to fewer journals [6], and the range of research available to readers is therefore increasingly limited. WJES open access policy, as described in the BioMed Central Open Access Charter [7], means that all articles become freely and universally accessible online, and so an author's work authors is disseminated to the widest possible audience. The journal's articles are also archived in PubMed Central [8], the US National Library of Medicine's full-text repository of life science literature, as well as in repositories at the University of Potsdam [9] in Germany, at INIST [10] in France and in e-Depot [11], the National Library of the Netherlands' digital archive of all electronic publications. Authors hold copyrights for their work and grant anyone the right to reproduce and disseminate the article [7].

*World Journal of Emergency Surgery *will defray the costs of publication from article-processing charges (APCs), because it does not have subscription charges for its content. Authors are asked to pay GBP750 if their research is accepted for publication. However, authors from resource-poor countries, who are unable to pay the APC for their manuscript can apply to the Editors-in-Chief, to have the APC waived [12]. Other authors, who belong to BioMed Central member organizations, will have the APC covered in full or part by the membership [13]. No charge is made for articles that are rejected after peer review.

We believe *World Journal of Emergency Surgery *will benefit clinical care and aid scientific research, and we hope you will support this progress by submitting your next research article to this open access journal.
